# Long-term follow-up after acute mercury poisoning-induced pneumonitis following cinnabar heating: A rare case report

**DOI:** 10.1097/MD.0000000000041013

**Published:** 2024-12-13

**Authors:** Khoa Nguyen-Dang, Anh-Thu Dau-Nguyen, Nguyen Tran-Ngoc, Ngoc Duong-Minh, Thong Dang-Vu, Sang Nguyen-Ngoc, Nam Vu-Hoai, Hung Le-Quoc

**Affiliations:** a Department of Internal Medicine, Faculty of Medicine, University of Medicine and Pharmacy at Ho Chi Minh City, Ho Chi Minh, Vietnam; b Department of Pulmonary Medicine, Cho Ray Hospital, Ho Chi Minh, Vietnam; c Department of Tuberculosis and Lung Diseases, Faculty of Medicine, University of Medicine and Pharmacy at Ho Chi Minh City, Ho Chi Minh, Vietnam; d Department of Tropical Medicine, Cho Ray Hospital, Ho Chi Minh, Viet Nam.

**Keywords:** chelation therapy, cinnabar, high-flow nasal cannula, mercury poisoning, methylprednisolone

## Abstract

**Rationale:**

Among 3 forms of mercury, elemental mercury vapor presents the highest threat due to its potential to cause acute pneumonitis. The management of acute mercury vapor poisoning remains unclear, particularly in acute lung injury. We present a case of mercury vapor poisoning resulting from the heating of cinnabar, successfully treated with high-dose corticosteroids and chelation therapy, and follow-up over 6 months.

**Patient concerns:**

A 47-year-old female patient was admitted to the Emergency Department due to dyspnea, chest tightness, and weakness following cinnabar heating.

**Diagnoses:**

Upon admission, she presented with tachypnea and respiratory failure. During the first 5 days, the respiratory failure rapidly progressed, requiring high-flow nasal cannula support, and showed no improvement with broad-spectrum intravenous (IV) antibiotics and 80 mg daily IV methylprednisolone. Total blood and urinary mercury levels were measured to confirm the diagnosis.

**Interventions:**

Upon confirmation of acute pneumonitis due to mercury vapor poisoning, the patient was administered high-dose methylprednisolone (500 mg IV per day) and chelation therapy, which led to subsequent improvement.

**Outcomes:**

Six months after discharge, the patient completely recovered, as evidenced by chest imaging and pulmonary function tests.

**Lessons:**

Heating elemental mercury can cause pneumonitis, leading to acute respiratory failure. A detailed history is crucial for diagnosis. High-dose methylprednisolone should be considered in patients who do not respond to lower doses. Patients should be monitored afterward to detect residual pulmonary fibrotic changes.

## 1. Introduction

Mercury exists in various forms in the environment, each with different impacts on human health. People can be exposed to mercury (elemental, inorganic, or organic) through multiple routes, including inhalation, ingestion, and dermal contact.^[1,2]^ This can lead to a wide spectrum of clinical presentations, which are often nonspecific and sometimes challenging to diagnose.^[1,3]^ Mercury poisoning through inhalation, particularly in the context of heating inorganic mercury compounds or elemental mercury, can lead to acute pneumonitis, rapidly progressive respiratory failure, acute respiratory distress syndrome, and death.^[1,4,5]^ This is the most severe manifestation of mercury poisoning.

Despite extensive knowledge about the chemical structure of mercury in the environment and numerous reports of mercury vapor-induced pneumonitis, significant gaps in evidence remain regarding this condition.^[3–8]^ Evidence is still lacking regarding the management of mercury vapor poisoning-induced acute pneumonitis, including the indications and effectiveness of high-dose systemic corticosteroids and chelation therapy.^[1,3,7,9]^ Furthermore, long-term monitoring of mercury-induced lung damage using chest computed tomography (CT) scans, pulmonary function tests (PFT), and diffusing capacity of the lung for carbon monoxide tests has not been widely reported.^[4,6,8]^ Consequently, treatment guidelines and the long-term prognosis for cases of acute pneumonitis due to inhaled mercury are not widely consensus and remain largely based on empirical treatment.

We report a case of mercury vapor poisoning from heating cinnabar at home, which resulted in acute pneumonitis and respiratory failure requiring high-flow nasal cannula (HFNC) support. The patient was successfully treated with high-dose methylprednisolone and chelation therapy. Fibrotic lung changes persisted for 3 months postexposure on chest CT scan. However, by the sixth month following discharge, the patient had fully recovered both chest imaging and lung function.

## 2. Case report

A 47-year-old female patient, with no previous medical history, was admitted to the Emergency Department of Cho Ray Hospital due to dyspnea, chest tightness, and weakness. Two days before admission, the patient purchased cinnabar (in powder form, orange in color) in a local traditional medicine pharmacy and burned it in a closed room to dispel evil spirits. At that time, 3 people were in that enclosed room, including the patient, the patient’s husband, and 1 child. Four hours after starting to heat the cinnabar, all individuals experienced a mild fever and weakness. However, another child in a different room did not show any symptoms. The patient had more severe symptoms, including dyspnea and chest tightness, as the patient spent the most time next to the burning cinnabar (the patient even covered herself with a blanket to inhale more vapor from the burning cinnabar).

At the Emergency Department, the patient presented with tachypnea (26 breaths per minute), a SpO_2_ of 86% on room air, and 96% with 6 L/minute of oxygen via nasal cannula. Lung auscultation revealed bilateral crackles. No dermatological, gastrointestinal, or neurological symptoms were noted, and examination of other organs did not reveal any abnormalities.

**Figure 1.**
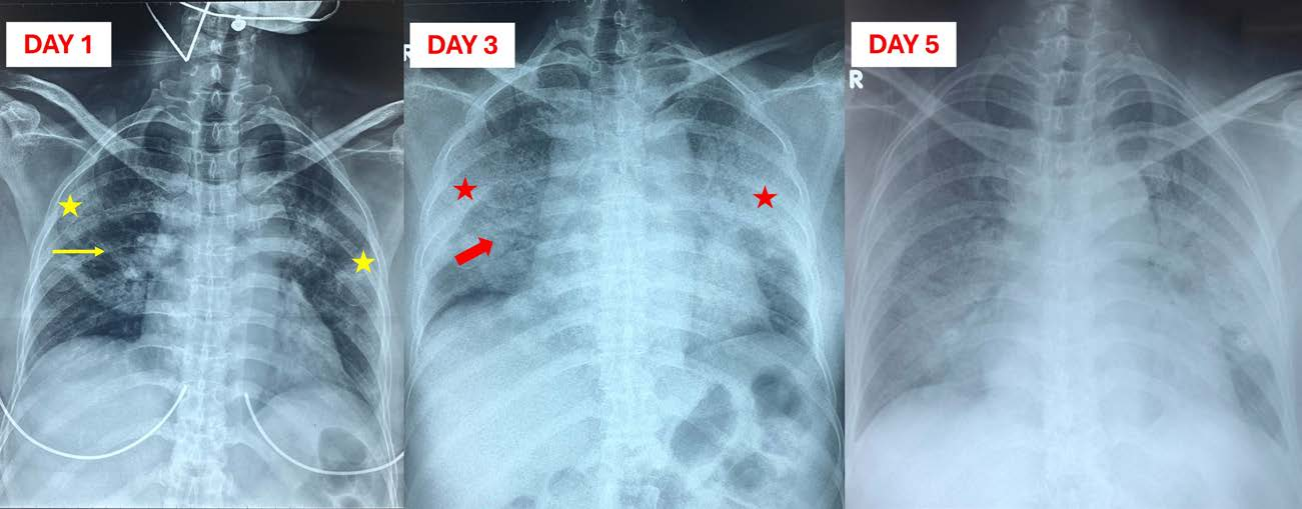
Chest X-ray from day 1 to day 5 postadmission. Day 1: Chest X-ray shows linear interstitial patterns (yellow line arrow) and focal consolidations (yellow star). Day 3: Lesions have progressed to bilateral lung consolidations (red arrow) with air-bronchogram signs (red arrow). Day 5: Consolidations observed on day 3 have further worsened, indicating a severe progression of the lung damage.

From the first to the fifth day of hospitalization, the patient’s respiratory failure rapidly worsened, progressing from oxygen via nasal cannula to requiring HFNC (with FiO_2_ increasing from 80% to 100% and flow rate of 40 L/minute) to maintain SpO_2_ above 94%. The patient was administered intravenous (IV) antibiotics, including moxifloxacin, meropenem, teicoplanin, and methylprednisolone (40 mg IV every 12 hours), along with other supportive treatments. Laboratory tests are presented in Table 1, showing leukocytosis (predominantly neutrophils) and increased serum transaminase levels. Chest X-ray revealed diffuse lung infiltration in both lung fields, with rapid progression after 5 days of treatment (Fig. 1).

**Table 1:** Laboratory results.

Test	Normal value	Day				
		1–2	4–5	8	11	15
WBC	4–10 G/L	**23.6**	**12.3**	**17.7**	**21.8**	**20.5**
Neutrophil	1.8–8.3 G/L	**21.5**	**10.1**	**13.9**	**17.7**	**16.2**
CRP	<6 mg/L	**229**	**90**			**6.2**
Procalcitonin	<0.5 ng/mL		0.07	0.02	0.02	
AST	9–48 U/L	38	**385**	**164**	**60**	
ALT	5–49 U/L	42	**118**	34	17	
Total serum bilirubin	0.2–1.0 mg/dL		0.56	0.58	0.58	
Serum creatinine	0.7–1.5 mg/dL	0.65	0.55	0.54	0.54	
Total blood mercury level	<18 µg/dL		**29.3**		7.7	
Urine mercury level			164		302	

Abnormal values are highlighted in bold.

ALT, alanine aminotransferase; AST, aspartate aminotransferase; CRP = C-reactive protein, WBC = white blood cells.

**Figure 2.**
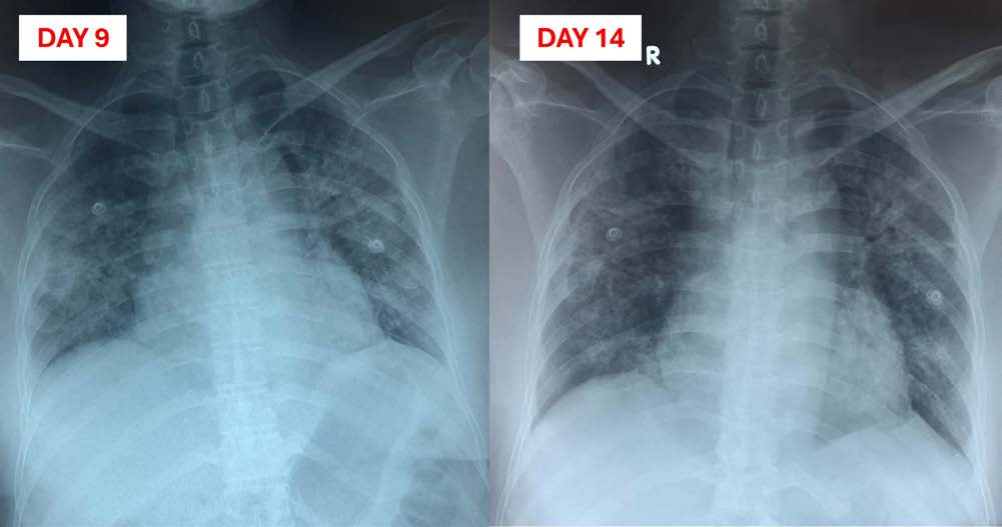
Chest X-ray from day 9 to day 14 postadmission shows significant reductions in lung consolidations compared to Figure 1.

From day 5 to day 18 after admission, following the patient’s report of using cinnabar (containing elemental mercury) and burning it indoors, total blood mercury levels and urinary mercury levels were measured. On day 5, after confirming acute pneumonitis due to mercury vapor poisoning and obtaining a low blood procalcitonin level (Table 1) with no response to 5 days of IV antibiotic treatment, all antibiotics were discontinued, and high-dose methylprednisolone therapy was initiated. The high-dose methylprednisolone regimen was as follows: 500 mg IV for the first 5 days, then 250 mg IV for the next 3 days, followed by 125 mg IV and tapering to 32 mg oral methylprednisolone daily. Dimercaptosuccinic acid (DMSA) was added orally at a dose of 10 mg/kg every 8 hours (600 mg every 8 hours) for the first 5 days, starting on day 8 of treatment due to its unavailability at our hospital. The DMSA dose was then reduced to 10 mg/kg every 12 hours (600 mg every 12 hours) for the following 14 days. After adjusting the treatment based on the diagnosis of acute pneumonitis due to mercury poisoning, the patient’s condition gradually improved, transitioning from HFNC to an oxygen mask at 8 L/minute on the eighth day after starting high-dose methylprednisolone. Chest X-ray showed improvement in bilateral lung fields (Fig. 2). The patient was discharged on day 18 postadmission.

**Figure 3.**
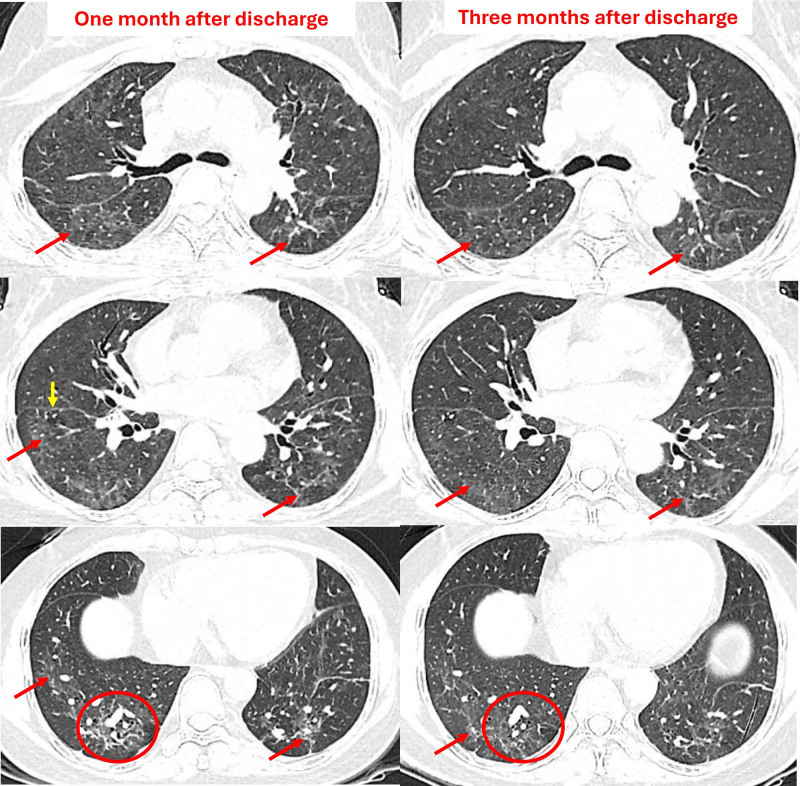
Chest computed tomography (CT) at 1 and 3-month follow-up. CT scans show scattered ground-glass opacities in both lungs, primarily in the lower lobes, accompanied by interlobular septal thickening (red arrow). There are also signs of interlobular fissure traction (yellow arrow), suggesting fibrotic changes in the lungs. Note the area of traction bronchiectasis (red circle). These lesions were significantly diminished on the CT scan taken 3 months later. CT = computed tomography.

The patient was followed up after discharge. Although there were no clinical symptoms, a chest CT scan at 1 and 3 months showed scattered ground-glass opacities in both lung fields, interlobular septal thickening, and traction of the interlobular fissure, suggesting fibrotic changes (Fig. 3). PFT was within normal limits, with vital capacity (VC) = 2.43 L (84%), forced vital capacity (FVC) = 2.38 L (82%), forced expiratory volume in 1 second (FEV_1_) = 2.31 L (96%), FEV_1_/VC = 95%, and diffusing capacity of the lung for carbon monoxide = 15.62 mL/mm Hg/min (*z* score = −1.52). A third chest CT scan taken 6 months postdischarge showed complete resolution of all previous lesions. After 6 months, the patient fully recovered regarding clinical symptoms, imaging findings, and lung function.

## 3. Discussion

Our case contributes 2 considerable points to the current literature: firstly, it presents a severe case of acute pneumonitis due to mercury vapor poisoning that was successfully treated with high-dose methylprednisolone and chelation therapy; secondly, it provides evidence of prolonged lung tissue damage following mercury vapor exposure, with a resolution period extending up to 6 months. However, due to the limitations of a single case report, we cannot draw any definitive conclusions regarding the treatment and management of acute pneumonitis caused by mercury vapor poisoning. Further studies are needed to clarify the unresolved issues surrounding this rare clinical presentation.

Cinnabar, composed of mercury sulfide, is an inorganic form of mercury.^[10]^ For centuries, cinnabar has been used as a traditional medicine in many Asian countries. When heated, cinnabar produces mercury vapor, which is easily absorbed through the respiratory tract.^[10,11]^ Mercury vapor crosses the alveolar-capillary barrier and enters the bloodstream, subsequently accumulating in the kidneys and central nervous system, leading to toxicity.^[1,12]^ Mercury poisoning through inhalation, particularly in the context of heating inorganic mercury compounds or elemental mercury, can lead to acute pneumonitis, rapidly progressive respiratory failure, acute respiratory distress syndrome, and death.^[1,4,5]^ In this case, other family members also demonstrated symptoms, and a thorough history revealed the burning of cinnabars, prompting consideration of acute pneumonitis due to mercury poisoning. However, due to the rarity of this condition and the mild symptoms in other family members, the patient initially dismissed this aspect, contributing to a delayed diagnosis.

Currently, there is no consensus on the treatment of acute pneumonitis due to mercury vapor poisoning. All available evidence is based on case reports, case series, or expert opinion.^[1,3,5–9]^ In this case, we will focus on discussing high-dose methylprednisolone and chelation therapy, which were key in contributing to the patient’s favorable prognosis.

High-dose IV methylprednisolone has been empirically used in severe, life-threatening cases of acute pneumonitis due to mercury vapor poisoning.^[4–6]^ However, the dosage of methylprednisolone varies significantly across reports, leading to uncertain evidence regarding this treatment approach. Bryan administered 500 mg of methylprednisolone IV every 12 hours for 2 days; although the patient’s respiratory failure improved rapidly, the patient eventually died due to persistent hypoxemia.^[5]^ Hammerling et al^[6]^ initially used 80 mg of methylprednisolone IV daily, but as the patient’s condition deteriorated, the dose was increased to 1 gram IV daily (the total duration of this higher dose was not reported). Hong used 500 mg of methylprednisolone IV daily for 3 days, followed by oral prednisolone (1 mg/kg) for the next 7 days.^[4]^ In our case, after monitoring that a daily dose of 80 mg of methylprednisolone IV was inadequate, we promptly switched to the following methylprednisolone regimen: 500 mg IV for the first 5 days, then 250 mg IV for the next 3 days, followed by 125 mg IV, and tapering to 32 mg oral methylprednisolone daily upon the patient’s discharge. There are 2 reasons supporting the usefulness of this high-dose and prolonged regimen. Firstly, an intermediate phase in the pathophysiology of acute mercury vapor-induced lung injury may occur 2 weeks after onset, characterized by rapid multi-organ dysfunction.^[4,6,8]^ Therefore, early cessation or reduction of corticosteroid dose may not prevent multi-organ failure in this intermediate phase.^[4]^ This hypothesis has been observed in Bryan’s report.^[5]^ Secondly, corticosteroids may prevent the progression of fibrotic changes in lung tissue, although this mechanism is not yet fully understood.^[4,6]^ Therefore, we suggest a prolonged and high-dose regimen of methylprednisolone in the treatment of acute pneumonitis due to mercury vapor poisoning.

Chelation therapy has long been used to treat heavy metal poisoning, including mercury.^[13]^ However, the indications for its use remain unclear. Experts recommend chelation therapy for patients showing symptoms of mercury poisoning.^[1]^ The Association of Clinical Biochemists advises chelation therapy for symptomatic patients with blood mercury levels > 100 µg/L or any patient with blood mercury levels > 200 µg/L.^[14]^ However, the actual usefulness of chelation therapy remains unclear.^[3,5,7,13]^ Dimercaptopropane sulfonate (DMPS) and DMSA are preferred because they do not risk redistributing mercury to the brain.^[1,7,13]^ In our case, chelation therapy contributed minimally to the patient’s recovery for 2 reasons. Firstly, chelation therapy is most effective when administered as early as possible, within minutes to hours after exposure, without waiting for blood mercury levels to confirm the diagnosis.^[6,7]^ In our report, DMSA was initiated on the fifth-day postadmission due to the unavailability of this drug in our region. Secondly, the role of chelation therapy in acute mercury-induced lung injury is uncertain because lung damage from mercury occurs rapidly after exposure, and reducing blood mercury levels does not impact the resolution of these pulmonary lesions.^[4,5]^ However, we prioritized using chelation therapy if available to reduce the patient’s overall body mercury burden.

Our case reveals that the patient displayed persistent pulmonary fibrotic and ground-glass opacities up to 3 months after mercury vapor exposure. This agrees with mercury’s half-life of approximately 60 days.^[1,5]^ Reports on PFTs or chest imaging for long-term follow-up in cases of acute mercury-induced lung injury are rare. Reported prolonged lesions include interstitial fibrosis, pulmonary granulomas, bronchiectasis, pulmonary fibrosis, interlobular septal thickening, and cysts.^[6,8]^ In our case, the patient’s lung lesions resolved slowly despite the absence of treatments such as corticosteroids or chelation therapy. More reports on persistent lung injury are needed to identify patients at risk for progressing to irreversible lung damage.

## 4. Conclusion

Heating elemental mercury can cause pneumonitis, leading to acute respiratory failure and death. A detailed history is crucial for diagnosis. High-dose methylprednisolone should be considered in patients who do not respond to lower doses. Patients should be monitored afterward to detect residual pulmonary fibrotic changes.

## Author contributions

**Conceptualization:** Khoa Nguyen-Dang, Anh-Thu Dau-Nguyen, Ngoc Duong-Minh, Thong Dang-Vu, Sang Nguyen-Ngoc, Nam Vu-Hoai, Hung Le-Quoc.

**Data curation:** Khoa Nguyen-Dang, Anh-Thu Dau-Nguyen, Ngoc Duong-Minh, Thong Dang-Vu, Sang Nguyen-Ngoc, Nam Vu-Hoai, Hung Le-Quoc.

**Formal analysis:** Anh-Thu Dau-Nguyen, Thong Dang-Vu.

**Funding acquisition:** Anh-Thu Dau-Nguyen, Thong Dang-Vu.

**Investigation:** Nguyen Tran-Ngoc, Ngoc Duong-Minh, Nam Vu-Hoai.

**Methodology:** Nguyen Tran-Ngoc, Ngoc Duong-Minh.

**Resources:** Hung Le-Quoc.

**Supervision:** Hung Le-Quoc.

**Validation:** Khoa Nguyen-Dang, Nam Vu-Hoai, Hung Le-Quoc.

**Visualization:** Khoa Nguyen-Dang, Nam Vu-Hoai, Hung Le-Quoc.

**Writing – original draft:** Khoa Nguyen-Dang, Anh-Thu Dau-Nguyen, Nguyen Tran-Ngoc, Ngoc Duong-Minh, Thong Dang-Vu, Sang Nguyen-Ngoc, Nam Vu-Hoai, Hung Le-Quoc.

**Writing – review & editing:** Khoa Nguyen-Dang, Ngoc Duong-Minh, Thong Dang-Vu, Sang Nguyen-Ngoc, Nam Vu-Hoai, Hung Le-Quoc.
